# Non-Invasive Assessment of Metabolic Dysfunction-Associated Steatotic Liver Disease and Cardiovascular Risk in Acromegaly Indicates Persistence of Cardiac Risks Despite Biochemical Disease Control

**DOI:** 10.3390/jcm14144822

**Published:** 2025-07-08

**Authors:** Yusuf Karadeniz, Melia Karakose

**Affiliations:** 1 Department of Internal Medicine, Division of Endocrinology and Metabolism, Karaman Training and Research Hospital, Karaman 70200, Türkiye; 2 Department of Internal Medicine, Division of Endocrinology and Metabolism, Faculty of Medicine, Necmettin Erbakan University, Konya 42090, Türkiye

**Keywords:** acromegaly, metabolic dysfunction-associated steatotic liver disease, fibrosis, cardiovascular risk, growth hormone

## Abstract

**Background/Objectives**: Acromegaly is associated with multiple metabolic comorbidities, but the relationship between disease activity and metabolic dysfunction-associated steatotic liver disease (MASLD) or cardiovascular risk remains unclear. This study aimed to assess the prevalence and severity of MASLD and liver fibrosis in patients with acromegaly relative to healthy controls and explore whether disease activity influences these parameters. We also evaluated cardiovascular risk indicators in acromegaly patients. **Methods**: A retrospective case-control study was conducted between 2000 and 2022, involving 58 acromegaly patients and 58 healthy controls. Patients were classified as active or in biochemical remission. MASLD was assessed using the fibrosis-4 (FIB-4) index, MASLD fibrosis score, body mass index, diabetes (BARD) score, the aspartate aminotransferase-to-platelet index (APRI), and the aspartate aminotransferase-to-alanine aminotransferase ratio. Cardiovascular evaluation included pulse wave velocity (PWV) and carotid intima–media thickness (CIMT). **Results**: The median age of the acromegaly group was 47.5 (39–57) years, compared to 42 (40–48) years in the control group (*p* = 0.041). APRI (*p* < 0.001), FIB-4 (*p* < 0.001), MASLD fibrosis score (*p* < 0.001), and BARD score (*p* < 0.001) were significantly higher in the acromegaly group. The prevalence of hepatic steatosis was also higher in the acromegaly group (*p* < 0.001). Diastolic blood pressure (*p* = 0.015) and PWV (*p* = 0.012) were significantly higher in the acromegaly group. **Conclusions**: Acromegaly patients have an increased risk of MASLD and fibrosis, but this risk is unassociated with disease activity. Similarly, cardiovascular risk parameters remain elevated regardless of disease activity. These findings suggest that the systemic effects of acromegaly may persist despite biochemical control.

## 1. Introduction

Acromegaly represents a rare endocrine disorder characterized by chronic growth hormone (GH) hypersecretion, most commonly originating from pituitary adenomas [1]. Epidemiological studies report a consistent annual incidence ranging from 3 to 11 cases per million person–years across diverse populations [2]. The persistent elevation of both GH and insulin-like growth factor-1 (IGF-1), characteristic of this condition, leads to progressive multisystem complications, including well-documented cardiovascular pathology, metabolic disturbances, and increased malignancy risk, all contributing to reduced quality of life and elevated mortality rates [1,3]. Among these systemic effects, the metabolic consequences—particularly insulin resistance and dyslipidemia—have received increasing attention due to their potential impact on hepatic pathophysiology [3].

The conceptualization of metabolic dysfunction-associated steatotic liver disease (MASLD) has evolved significantly in recent years, replacing the previous NAFLD terminology to better reflect the central role of metabolic dysfunction in disease pathogenesis [4]. Current epidemiological data estimate that approximately 25% of the global population meets the diagnostic criteria for MASLD, establishing it as the most prevalent chronic liver condition worldwide [5,6]. The disease spectrum encompasses simple steatosis, steatohepatitis, and progressive fibrosis, with disease progression substantially increasing risks of cirrhosis and hepatocellular carcinoma [5,6]. While liver biopsy remains the diagnostic gold standard, its invasive nature has driven widespread adoption of non-invasive assessment tools, including the AST-to-platelet ratio index (APRI), fibrosis-4 index (FIB-4), MASLD fibrosis score, and BARD score in clinical practice [4,7]. Ultrasonography has similarly emerged as a valuable first-line imaging modality for steatosis detection and monitoring [4,7].

The interplay between GH/IGF-1 axis dysregulation and hepatic lipid metabolism presents a particularly intriguing area of investigation. Experimental models have demonstrated that GH exerts direct lipolytic effects while simultaneously inducing insulin resistance through complex mechanisms [3,8]. These seemingly contradictory actions create a metabolic paradox where GH excess could theoretically both promote and protect against hepatic steatosis. Clinical observations have further complicated this picture, with some studies suggesting GH deficiency may actually represent a more significant risk factor for hepatic fat accumulation than GH excess [3,5,8]. The recent work by Fellinger et al. [9] provided mechanistic insights by demonstrating enhanced hepatic ATP synthesis in active acromegaly, potentially explaining reduced lipid accumulation despite systemic insulin resistance.

Previous clinical investigations examining the acromegaly–MASLD relationship have been limited by several methodological challenges. Most notably, studies have frequently employed small sample sizes and inconsistent approaches to controlling for key metabolic confounders, such as age, body composition, and comorbid conditions [9,10,11,12,13,14]. Additionally, few studies have systematically compared active versus biochemically controlled acromegaly patients, leaving important questions about disease activity effects unanswered. These limitations in the existing evidence base highlight the need for more rigorous examinations of hepatic metabolic consequences in acromegaly.

In this study, we aimed to evaluate the prevalence and severity of MASLD in acromegaly patients using non-invasive tests and to compare characteristics with a healthy control group. Furthermore, by comparing active and biochemical remission-phase patients, we aimed to determine whether disease activity influences the occurrence and severity of MASLD. Secondarily, we also sought to assess cardiovascular risk factors in patients with acromegaly.

## 2. Materials and Methods

### 2.1. Setting and Population

**Figure 1:**
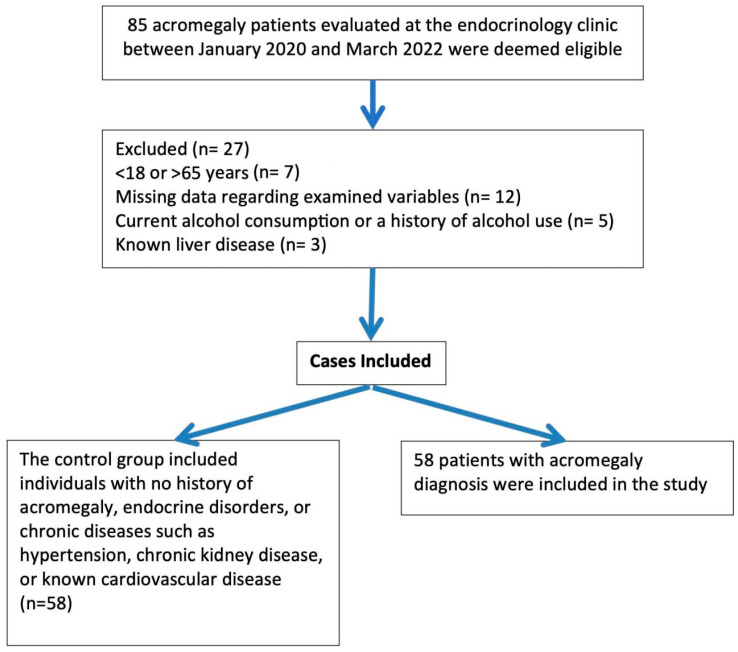
Flow chart of the study.

This retrospective case-control study was conducted at the Department of Endocrinology, Necmettin Erbakan University Faculty of Medicine. Ethical approval, in accordance with the Declaration of Helsinki, was obtained from the Necmettin Erbakan University Non-Drug and Non-Medical Device Research Ethics Committee (Decision date: 13 May 2022, decision No. 2022/3781). The patient group consisted of eligible individuals diagnosed with acromegaly at the endocrinology outpatient clinic between January 2000 and March 2022. The control group included individuals with no history of acromegaly, endocrine disorders, or chronic diseases such as hypertension, chronic kidney disease, or known cardiovascular disease. The exclusion criteria for both groups included being younger than 18 or older than 65 years, missing data regarding the examined variables, current alcohol consumption or a history of alcohol use, and known liver disease (Figure 1) (Table 1). Eligible control subjects were randomly selected using IBM SPSS version 25.0 from individuals without chronic disease who had undergone routine outpatient evaluations. Although not matched on a one-to-one basis, the controls were screened to exclude chronic illnesses and major metabolic comorbidities.

According to the descriptive statistics (effect size = 1.475) in the study by Saleem et al. [15], a sample size of 22 achieves 90% power at the two-sided 0.05 significance level. Power analysis was performed using G*Power 3.1.9.4 software.

### 2.2. Data Collection

Demographic data, including age and sex, anthropometric measurements, smoking status, comorbidities, laboratory findings, liver imaging results, and cardiovascular measurements, were obtained retrospectively from the hospital’s electronic medical records.

#### 2.2.1. Diagnosis and Classification of Acromegaly

Acromegaly was diagnosed based on clinical findings and biochemical criteria, including elevated IGF-1 levels adjusted for age and sex, and failure to suppress GH levels below 1 ng/mL (or 0.4 ng/mL with ultrasensitive assays) following a 75 g oral glucose tolerance test (OGTT) [16,17]. Magnetic resonance imaging (MRI) was used to assess the presence and size of pituitary adenomas. Diagnosis and therapeutic management were based on the international consensus guidelines published between 2000 and 2022, including those from the Endocrine Society, the Pituitary Society, and the European Society of Endocrinology.

Disease activity was classified based on IGF-1 levels and GH measurements [13,18]. Active disease was defined as persistently elevated IGF-1 levels and/or GH > 1 ng/mL despite treatment. Biochemical remission was defined as normalized IGF-1 levels and GH suppression (<1 ng/mL) after OGTT in patients who had undergone surgical, medical, or radiotherapy treatment. Newly diagnosed patients were defined as those who had not yet received treatment at the time of enrollment. Adenomas were classified based on MRI findings. Macroadenomas were defined as pituitary adenomas with a maximum diameter of ≥10 mm, while microadenomas were defined as pituitary adenomas with a maximum diameter of <10 mm. Disease duration was calculated as the time from symptom onset, as reported by the patient, to the date of diagnosis. If symptom onset was unclear, the duration was based on the earliest available medical records indicating biochemical or radiological evidence of acromegaly.

#### 2.2.2. Anthropometric Measurements

Body mass index (BMI) was calculated using the formula weight (kg) divided by the height squared (m^2^). Obesity was defined as BMI ≥ 30 kg/m^2^, while extreme obesity was defined as BMI ≥ 40 kg/m^2^. Waist circumference was measured at the midpoint between the lowest rib and the iliac crest using a flexible, non-stretchable measuring tape while the participants stood and breathed normally. Hip circumference was measured at the widest part of the buttocks using the same tape, ensuring it remained parallel to the floor. The waist-to-hip ratio (WHR) was calculated by dividing the waist circumference by the hip circumference. WHR values were classified as ‘normal’ or ‘high’ based on established cutoff values, with ‘high’ defined as >0.90 for men and >0.85 for women. Waist circumference was recorded using standard clinical protocols available at the time of assessment. However, temporal variation in the documentation practices may exist.

#### 2.2.3. Laboratory Measurements and Related Variables

All biochemical and hematological analyses were performed in a certified clinical laboratory accredited by international quality control programs. Blood samples were collected after an overnight fast and processed using standardized protocols. For acromegaly patients, samples were obtained at diagnosis and follow-up. For the control group, samples were collected during routine outpatient visits. IGF-1 was measured using a chemiluminescent immunoassay (CLIA) and adjusted for age and sex. GH was determined by CLIA, with both random and stimulated (OGTT) values recorded. IGF-1 peak was defined as the highest IGF-1 level measured during diagnostic evaluation, while GH peak was defined as the highest GH level measured during OGTT. Glucose was measured using the hexokinase method, and insulin levels were assessed by CLIA. Glycated hemoglobin (HbA1c) was measured using high-performance liquid chromatography. C-reactive protein (CRP) was quantified using an immunoturbidimetric assay. Lipid profile parameters, including total cholesterol, high-density lipoprotein cholesterol (HDL-C), low-density lipoprotein cholesterol, and triglycerides, were analyzed using enzymatic colorimetric methods. Liver enzymes, including AST, alanine aminotransferase (ALT), gamma-glutamyl transferase, and alkaline phosphatase, were measured using enzymatic colorimetric assays. Albumin was measured using the bromocresol green method. Hematological parameters, including the white blood cell count; neutrophil, lymphocyte, monocyte, and platelet counts; red cell distribution width coefficient of variation; and hemoglobin level, were measured using an automated hematology analyzer. Due to the extended study period (2000–2022), the assay methods may have varied slightly over time. CLIA was the standard for GH and IGF-1 measurement during the later years of the study, but retrospective uniformity could not be ensured.

#### 2.2.4. Calculation of Insulin Resistance and Liver Fibrosis Scores

Homeostatic model assessment of insulin resistance (HOMA-IR) was calculated using the formula HOMA-IR = (Fasting Insulin (μU/mL) × Fasting Glucose (mg/dL))/405 [19]. The APRI was calculated as APRI = (AST (U/L)/Upper Limit of Normal AST) × 100/Platelet Count (10^3^/µL) [7]. The FIB-4 index was determined using the formula FIB-4 = (Age (years) × AST (U/L))/(Platelet Count (10^9^/L) × √ALT (U/L)) [7]. The MASLD fibrosis score was calculated as −1.675 + (0.037 × Age) + (0.094 × BMI) + (1.13 × Diabetes (1 = yes, 0 = no)) + (0.99 × AST/ALT Ratio) − (0.013 × Platelet Count) − (0.66 × Albumin) [7]. The BARD score was derived based on body mass index (BMI) ≥ 28 kg/m^2^ (1 point), AST/ALT ratio ≥ 0.8 (1 point), and diabetes mellitus (1 point), with a total score ranging from 0 to 3 [7].

#### 2.2.5. Liver Ultrasonography Assessment

Liver ultrasonography was performed using a high-resolution ultrasound device to evaluate hepatic echogenicity. Fatty liver was classified as normal (no increased echogenicity), grade 1 (mild increased echogenicity with normal visualization of the diaphragm and intrahepatic vessels), and grade 2 (moderate increased echogenicity with slightly impaired visualization of intrahepatic structures) [20].

#### 2.2.6. Cardiovascular Measurements and Indices

Mean arterial pressure was calculated as (Systolic Blood Pressure + 2 × Diastolic Blood Pressure)/3 [21]. Pulse pressure was defined as the difference between the systolic and diastolic blood pressure [22]. Heart rate was measured using an automated oscillometric device. Carotid intima–media thickness (CIMT) was assessed using high-resolution B-mode ultrasonography at the far wall of the common carotid artery. Augmentation pressure was determined as the difference between the second and first systolic peaks of the central arterial pressure waveform. The augmentation index was calculated as (Augmentation Pressure/Pulse Pressure) × 100 [23]. Pulse wave velocity (PWV) was measured using applanation tonometry by recording the pulse transit time between the carotid and femoral arteries.

### 2.3. Statistical Analysis

All analyses were conducted using IBM SPSS version 25.0 (IBM Corp., Armonk, NY, USA), with the classical *p* < 0.05 threshold accepted to show statistical significance. The Kolmogorov–Smirnov (Lilliefors correction) test was used to examine the conformity of the variables to normal distribution. For continuous variables, descriptive statistics were presented using the mean ± standard deviation for those that were normally distributed, and with median (25th percentile–75th percentile) for those with non-normal distribution. Continuous variables meeting normal distribution assumptions were analyzed using Student’s *t* test, while the Mann–Whitney U test was used when the assumptions were not met. Absolute and relative frequencies were used to summarize the categorical variables. Categorical variables were analyzed using chi-square tests or Fisher’s exact test (including its Freeman–Halton extension). Multivariable logistic regression analyses were performed to determine significant factors independently associated with acromegaly. Statistically significant variables according to univariate analysis results were included in the logistic regression analysis. Pearson or Spearman correlation coefficients were calculated to evaluate the directional relationships between the continuous variables, such as IGF-1, GH, and MASLD markers.

## 3. Results

The study included 58 patients diagnosed with acromegaly and 58 healthy controls. The median age was 47.5 years (39–57) in the acromegaly group and 42 years (40–48) in the control group, with the acromegaly group being significantly older (*p* = 0.041). The proportion of females was 62.07% in the acromegaly group and 67.24% in the control group, with no significant difference in sex distribution between the groups (*p* = 0.698). Smoking (*p* = 0.003), hypertension (*p* < 0.001), diabetes (*p* < 0.001), cardiovascular disease (*p* < 0.001), and the frequency of high WHR (*p* = 0.037) were significantly more common in patients with acromegaly. Fasting glucose (*p* = 0.002), HbA1c (*p* < 0.001), and triglyceride levels (*p* = 0.014) were significantly higher in the acromegaly group. Among liver enzymes, AST (*p* < 0.001) and ALP (*p* = 0.003) levels were significantly elevated in the acromegaly group, whereas albumin (*p* < 0.001) and hemoglobin (*p* = 0.011) levels were higher in the controls. The APRI (*p* < 0.001), FIB-4 (*p* < 0.001), and MASLD fibrosis scores (*p* < 0.001) were significantly higher in the acromegaly group, and hepatic steatosis was more prevalent in this group (*p* < 0.001). The diastolic blood pressure (*p* = 0.015), augmentation index (*p* = 0.005), and pulse wave velocity (*p* = 0.012) were significantly higher in the acromegaly group (Table 2).

According to multivariable logistic regression analysis results, high height (OR: 1.268, 95% CI: 1.019–1.578, *p* = 0.033), high glucose (OR: 1.113, 95% CI: 1.000–1.240, *p* = 0.049), and high MASLD fibrosis score (OR: 305.607, 95% CI: 5.635–16574.949, *p* = 0.005) were independently associated with acromegaly (Table 3). Other variables included in the analysis, including age, waist to hip ratio, HbA1c, triglyceride, AST, ALP, albumin, hemoglobin, platelet, APRI, FIB-4, BARD score, liver USG, diastolic blood pressure, augmentation pressure, augmentation index and pulse wave velocity, were found to be non-significant (*p* > 0.05 for all).

Among the patients with acromegaly, 24 (41.4%) were classified as having active disease, while 26 (44.8%) were in biochemical remission. The remaining eight patients (13.8%) were not included in this analysis due to the lack of sufficient data to determine disease activity. There was no significant difference between these groups in terms of age (*p* = 0.984) or sex (*p* = 0.321). ALT levels were significantly higher in the biochemical remission group (*p* = 0.015). However, the IGF-1 (*p* < 0.001) and GH (*p* < 0.001) levels were significantly higher in the active disease group. No other significant differences were observed between the groups, including APRI (*p* = 0.085), FIB-4 (*p* = 0.478), MASLD fibrosis score (*p* = 0.942), BARD score (*p* = 0.871), and liver ultrasonography findings (*p* = 0.273) (Table 4).

Finally, an examination of the relationships between examined parameters and scores showed no significant correlations between FIB-4, MASLD fibrosis score, BARD score, or liver ultrasonography findings and IGF-1, GH, IGF-1 peak, or GH peak levels (Table 5).

## 4. Discussion

The relationship between MASLD and acromegaly remains unclear due to the complex metabolic effects of GH [4]. GH influences energy metabolism by stimulating gluconeogenesis and glycogenolysis in the liver and muscle, while its lipolytic activity causes a release of free fatty acids (FFA) from adipose tissue [4]. Both insulin resistance and elevated FFA levels are well-established contributors to MASLD pathogenesis [3,24]. However, the increased lipolysis observed in acromegaly may facilitate the utilization of fatty acids in extrahepatic tissues, thereby preventing hepatic lipid accumulation and potentially exerting a protective effect against MASLD [9]. Considering this potentially paradoxical impact, we aimed to determine whether MASLD was more prevalent in acromegaly patients, assess the risk of hepatic fibrosis, and clarify the uncertain relationship between MASLD and GH/IGF-1 levels. Our findings revealed that acromegaly patients exhibited significantly higher APRI, FIB-4, MASLD fibrosis score, and BARD score values compared to controls. However, only the MASLD fibrosis score remained independently associated with acromegaly in multivariate adjustments. Moreover, the prevalence of grade 1 and grade 2 hepatic steatosis, as determined by ultrasonography, was significantly higher in the acromegaly group. However, none of these non-invasive parameters correlated with IGF-1 or GH levels.

Previous studies have reported conflicting results regarding the impact of acromegaly on hepatic steatosis and fibrosis. Eroğlu et al. found higher BARD scores in active acromegaly patients despite lower visceral adipose tissue, while controlled patients showed a higher TyG index and a greater MASLD risk compared to controls. Interestingly, MASLD patients had lower GH and IGF-1 levels [4]. Similarly, other studies have shown increased hepatic steatosis and fibrosis in acromegaly [15], with 83.8% of newly diagnosed patients presenting an elevated hepatic steatosis index, independent of GH, IGF-1, or metabolic markers [1]. Conversely, Fellinger et al. reported lower hepatocellular lipid and unsaturated-to-saturated fatty acid ratios, but higher hepatic ATP synthesis in active acromegaly, suggesting that GH excess may prevent lipid accumulation via enhanced ATP production [9]. Despite insulin resistance, reduced hepatic lipid content in acromegaly may reflect increased lipid oxidation [10]. Another study confirmed lower intrahepatic lipids but higher fasting insulin in active acromegaly [11]. Acromegaly has also been linked to lower hepatic steatosis, higher liver stiffness, and elevated IGF binding protein 7 and cytokeratin 18 levels, with fibrosis being more common and hepatic steatosis inversely related to GH levels [14,25]. Altered fat distribution has also been noted, with lower visceral and subcutaneous fat but increased intermuscular fat in active disease [13]. In GH-deficient patients with non-functioning pituitary adenomas, MASLD prevalence was twice as high as in non-deficient individuals [26]. Nguyen et al. found increased liver fat and MASLD prevalence in pituitary disease, with liver fat inversely correlated with IGF-1. Age, BMI, and IGF-1 were independent predictors [27]. However, Hu et al. showed a non-linear relationship between IGF-1 and MASLD [28].

While some findings suggest an increased risk of hepatic steatosis and fibrosis in acromegaly, others indicate a protective effect of GH against lipid accumulation in the liver, which complicates the assessment of potential bidirectional associations between acromegaly and MASLD. Our study demonstrated that acromegaly patients have higher non-invasive fibrosis scores and a greater prevalence of hepatic steatosis than controls, aligning with the majority of the literature. However, GH and IGF-1 levels did influence MASLD development in acromegaly. The discrepancies in previous research may be attributed to differences in study design, patient characteristics, and the metabolic status of acromegaly patients (active vs. controlled disease).

Acromegaly patients can be classified as active or in biochemical remission based on GH and IGF-1 levels. The impact of disease activity on metabolic and organ complications, particularly liver health, remains an important area of investigation. Active acromegaly is characterized by a unique metabolic profile, including reduced adiposity and increased insulin resistance. However, following surgical treatment, adiposity increases while insulin resistance declines [29]. In other populations, depot-specific fat distribution and ectopic lipid accumulation are key determinants of insulin resistance, but this relationship is not well established in acromegaly. Furthermore, the changes in hepatic lipid content following treatment remain unclear [29].

Another aim of our study was to assess MASLD prevalence and severity in active and biochemical remission-phase acromegaly patients. This comparison is crucial to determine whether disease control influences the risk of MASLD and fibrosis. Unlike Eroğlu et al. [4], we found no difference in MASLD prevalence or fibrosis scores between active and remission-phase patients, suggesting that disease control may not modulate hepatic risk [4]. However, there exists evidence showing no change in hepatic lipid content following transsphenoidal surgery for active acromegaly [10]. A prospective study showed that acromegaly treatment could trigger increased intrahepatic lipid, abdominal, and thigh fat mass, while muscle mass decreased [11], with support from case studies [12]. In an analysis of pre- and post-treatment (surgical) acromegaly patients, decreased levels of GH, IGF-1, and insulin resistance were detected, while visceral and subcutaneous fat mass increased [13]. However, it appears that increases in visceral fat, subcutaneous fat, and hepatic lipid mass can persist in the long term after surgical treatment [29]. Medical treatments also impact the metabolic dynamics of patients with acromegaly. Somatostatin receptor ligand therapy for 12 months yielded significantly lower levels of GH and IGF-1, and 58% of patients achieved biochemical disease control. At baseline, 19 patients had ultrasonographically confirmed steatosis, and 45.1% exhibited an elevated hepatic steatosis index. Post-treatment, HOMA-IR significantly decreased, the Insulin Sensitivity Index (Matsuda) score increased, and the Hepatic Steatosis Index scores declined [1]. Multiple studies have demonstrated that individuals with GH deficiency are more prone to developing hepatic steatosis [30,31]. Additionally, some studies have reported lower basal and stimulated GH levels in MASLD patients [8,32]. Two small-scale studies have suggested that recombinant GH therapy may improve hepatic steatosis [5,6].

Our findings suggest that MASLD prevalence and fibrosis risk do not differ significantly between active and biochemical remission-phase acromegaly patients. This contrasts with previous studies indicating that active disease may confer a protective effect against MASLD due to elevated GH levels [4]. However, other reports suggest that hepatic lipid content remains unchanged after surgical treatment, while body fat distribution shifts toward increased adiposity [10,11,12,13,29]. Although GH deficiency is associated with a higher risk of MASLD, and GH replacement therapy may have potential benefits [5,6,30,31], the direct impact of GH and IGF-1 on hepatic lipid metabolism remains unclear. Given these inconsistencies, future longitudinal studies with larger cohorts are needed to clarify the complex interplay between acromegaly, disease control, and MASLD progression.

Acromegaly leads to notable cardiovascular alterations [33]. Patients have increased risks of insulin resistance, diabetes, dyslipidemia, and hypertension, all contributing to heightened cardiovascular risk [18,34]. Acromegalic cardiomyopathy, marked by biventricular hypertrophy in up to 90% of long-standing cases, can progress to heart failure [33]. While cardiac effects are well studied, vascular involvement is less understood. In our study, diastolic blood pressure and pulse wave velocity (PWV) were significantly higher in acromegaly patients than in the controls, whereas the augmentation pressure and index were lower. No significant differences were observed between active and remission-phase patients, supporting previous findings [33]. Winhofer et al. reported greater systolic function and hypertrophy in active acromegaly, with more pericardial fat in controlled cases, suggesting ectopic cardiac fat may not drive morbidity [10]. Similarly, Paisley et al. found a higher PWV in acromegaly but no differences in CIMT or correlations with IGF-1, disease activity, or duration [35]. Other studies also report increased arterial stiffness and CIMT [36,37]. Our findings of elevated diastolic pressure and arterial stiffness align with these reports [18,33,34]. The lack of differences between disease phases suggests that biochemical control does not reverse cardiovascular changes [33,35]. A possible explanation is the older median age in the acromegaly group compared to controls, since PWV increases with age [38]. While our findings support previous data, the interpretation of PWV and other vascular parameters should be made cautiously. Given the well-established age dependence of PWV, the observed vascular differences may be at least partially attributable to the age discrepancy, potentially leading to an overestimation of cardiovascular risk attributed to acromegaly. Moreover, the persistence of elevated vascular markers in patients with biochemical remission raises an important clinical question: do these findings indicate irreversible structural changes in the vasculature caused by long-standing GH/IGF-1 excess, or do they reflect changes that are reversible but with a slow trajectory? This distinction has significant implications for patient management and risk stratification. Future longitudinal studies with repeated vascular assessments are necessary to determine whether biochemical control leads to a gradual normalization of the vascular parameters or whether some alterations persist as residual risk markers.

Our study has several strengths. Compared to similar studies, our sample size is relatively larger, allowing for a more robust analysis. Additionally, we conducted comparisons both between acromegaly patients and healthy controls and between active and biochemical remission-phase acromegaly patients. However, certain limitations should be acknowledged. The retrospective design inherently limits the ability to establish causal relationships. As a single-center study, the external validity of our findings may be restricted. Age matching was not performed between the acromegaly and control groups, which may have influenced some of our results, particularly those related to vascular parameters. The assessment of hepatic steatosis was based solely on non-invasive laboratory scores. One important limitation we need to acknowledge is the age gap between our patient and control groups (our acromegaly patients averaged 47.5 years vs. 42 years in controls). This 5.5-year difference, while seemingly small, was statistically significant and could have affected our results—especially for measurements like pulse wave velocity and arterial stiffness that naturally increase with age. This means we might be overestimating some of the cardiovascular risks we attributed to acromegaly. Additionally, detailed treatment data were not available, which prevented therapy-based assessments. Moreover, the control group was not matched for age, waist-to-hip ratio, or comorbidities—factors known to influence hepatic and cardiovascular outcomes. This introduces potential confounding and may limit causal interpretation. A key limitation is the lack of detailed treatment data, including drug types and durations. As medical therapies for acromegaly may variably impact hepatic and cardiovascular outcomes, this restricts the interpretation of the disease control effects. Finally, while chronic diseases were excluded from the control group, they could not be excluded in the acromegaly group due to their high prevalence in these patients, which was likely to have impacted cardiac and metabolic parameters. An additional limitation is the lack of routine screening for common comorbidities like celiac disease and other endocrine disorders, which may independently cause hepatic steatosis and liver enzyme elevation. While the prevalence of celiac disease in acromegaly remains unknown, its exclusion—particularly in patients with persistent transaminase elevation—could improve the specificity of hepatic findings [39]. We recommend a systematic evaluation of such confounders in future studies. Finally, emerging research suggests that broader metabolic interactions in acromegaly may extend beyond traditional pathways, including potential gut microbiota alterations [39] and microRNA-mediated regulation of metabolic processes [40,41]. While these mechanisms were not examined in our study, they represent promising avenues for understanding the systemic metabolic effects of GH/IGF-1 dysregulation.

## 5. Conclusions

The findings of this comprehensive study provide important insights into the complex metabolic and cardiovascular manifestations of acromegaly. Our data demonstrate that patients with acromegaly exhibit significantly higher prevalence of MASLD and more advanced hepatic fibrosis scores compared to healthy controls, as assessed through multiple non-invasive measures including APRI, FIB-4, MASLD fibrosis score, and BARD score. These hepatic alterations appear independent of disease activity status, persisting even in biochemically controlled patients, suggesting potential long-term metabolic consequences of GH/IGF-1 axis dysregulation that may not fully normalize with current treatment approaches.

The cardiovascular assessment similarly revealed persistent vascular changes in acromegaly patients, characterized by increased arterial stiffness and elevated diastolic blood pressure. Notably, these parameters showed no significant improvement in patients achieving biochemical remission, raising important questions about the reversibility of long-standing vascular remodeling in this population. The observed age discrepancy between groups, while statistically adjusted for in our analyses, warrants consideration in interpreting these vascular findings and highlights the need for age-matched prospective studies.

## Figures and Tables

**Table 1:** Study inclusion and exclusion criteria.

	Acromegaly Group	Control Group
Inclusion	Diagnosis of acromegaly (confirmed by IGF-1/GH levels and pituitary imaging)	Age 18–65 years
	Age 18–65 years	No history of endocrine disorders
	Treatment-naïve or currently treated patients	No chronic diseases (HT, CKD, CVD)
Exclusion	Age <18 or >65 years	Age <18 or >65 years
	Missing key data	Missing key data
	Alcohol consumption (current/history)	Alcohol consumption (current/history)
	Known liver disease	Known liver disease
		Any chronic metabolic condition

Abbreviations: CVD: cardiovascular disease; CKD: chronic kidney disease; GH: growth hormone; HT: hypertension; IGF-1: insulin-like growth factor 1.

**Table 2:** Summary of demographics and laboratory measurements with regard to groups.

	Groups		
	Acromegaly (*n* = 58)	Control (*n* = 58)	*p*
Age	47.5 (39–57)	42 (40–48)	**0.041 ^‡^**
Sex			
Female	36 (62.07%)	39 (67.24%)	0.698 ^§^
Male	22 (37.93%)	19 (32.76%)	
Height, cm	165 (161–174)	162 (157–168)	**0.043 ^‡^**
Weight, kg	87.5 (73–98)	80 (71–97)	0.224 ^‡^
Body mass index, kg/m^2^	30.61 (27.16–34.67)	30.25 (26.29–35.64)	0.971 ^‡^
Obesity	31 (53.45%)	30 (51.72%)	0.852 ^§^
Extreme obesity	4 (6.90%)	10 (17.24%)	0.154 ^§^
Waist circumference, cm	91.00 ± 12.31	92.16 ± 15.02	0.651 ^†^
Hip circumference, cm	102 (97–109)	105 (98–118)	0.200 ^‡^
Waist to hip ratio	0.88 ± 0.06	0.85 ± 0.08	0.058 ^†^
Normal	29 (50.00%)	41 (70.69%)	**0.037 ^§^**
High	29 (50.00%)	17 (29.31%)	
Smoking	9 (15.52%)	0 (0.00%)	**0.003 ^#^**
Hypertension	22 (37.93%)	0 (0.00%)	**<0.001 ^§^**
Diabetes mellitus	17 (29.31%)	0 (0.00%)	**<0.001 ^§^**
Cardiovascular disease	29 (50.00%)	0 (0.00%)	**<0.001 ^§^**
Disease activity			
Active	24 (41.38%)	-	-
Biochemical remission	26 (44.83%)	-	
Newly diagnosed	8 (13.79%)	-	
Type of adenoma			
Macroadenoma	45 (77.59%)	-	-
Microadenoma	13 (22.41%)	-	
Duration of disease, years	8 (1–14)	-	-
IGF-1	240.1 (155–348)	-	-
GH	1.48 (0.49–4.00)	-	-
IGF-1, peak	727.39 ± 347.09	-	-
GH, peak	6.08 (3.72–21.62)	-	-
Glucose	100.7 (92.3–120.1)	93.95 (88.8–99.4)	**0.002 ^‡^**
Insulin	9.86 (6.70–15.91)	11.85 (8.24–16.82)	0.131 ^‡^
HOMA-IR	2.48 (1.74–3.81)	2.59 (1.89–3.79)	0.623 ^‡^
HbA1c	5.9 (5.5–6.3)	5.4 (5.1–5.6)	**<0.001 ^‡^**
CRP	1.67 (0.67–3.54)	2.31 (1.02–3.99)	0.389 ^‡^
Total cholesterol	201.57 ± 33.74	190.36 ± 31.69	0.069 ^†^
HDL-C	47.49 ± 12.57	47.19 ± 10.14	0.886 ^†^
LDL-C	115.32 ± 32.22	112.17 ± 28.42	0.579 ^†^
Triglyceride	175.0 (132.9–252.0)	153.65 (87.8–208.1)	**0.014 ^‡^**
AST	25 (21–30)	16.35 (14–20)	**<0.001 ^‡^**
ALT	16 (12.2–20)	17.45 (14.1–23.2)	0.210 ^‡^
GGT	17 (11–24.35)	17 (12–23)	0.874 ^‡^
ALP	79 (66.5–95)	68 (55–82)	**0.003 ^‡^**
Albumin	4.13 ± 0.42	4.53 ± 0.27	**<0.001 ^†^**
WBC (×10^3^)	6.71 ± 1.80	6.82 ± 1.48	0.710 ^†^
Neutrophil (×10^3^)	3.54 (3.00–4.46)	3.76 (3.02–4.65)	0.540 ^‡^
Lymphocyte (×10^3^)	2.15 ± 0.54	2.23 ± 0.51	0.432 ^†^
Monocyte (×10^3^)	0.47 (0.39–0.55)	0.42 (0.37–0.50)	0.089 ^‡^
RDW-CV	13.5 (13–14.3)	13.4 (13–14)	0.759 ^‡^
Hemoglobin	13.17 ± 1.27	13.83 ± 1.47	**0.011 ^†^**
Platelet (×10^3^)	199.59 ± 42.70	275.98 ± 63.09	**<0.001 ^†^**
APRI	0.32 (0.27–0.39)	0.15 (0.13–0.19)	**<0.001 ^‡^**
FIB-4	1.46 (1.19–1.75)	0.60 (0.50–0.81)	**<0.001 ^‡^**
MASLD fibrosis score	0.11 ± 1.14	−2.78 ± 0.89	**<0.001 ^†^**
BARD score	3 (3–4)	2.5 (1–3)	**<0.001 ^‡^**
Liver USG			
Normal	8 (21.62%)	31 (81.58%)	**<0.001**
Grade 1	21 (56.76%)	6 (15.79%)	
Grade 2	8 (21.62%)	1 (2.63%)	
Systolic blood pressure	123 (114–136)	120.5 (112–130)	0.162 ^‡^
Diastolic blood pressure	84.81 ± 13.57	78.74 ± 12.77	**0.015 ^†^**
Mean arterial pressure	95 (89–108.33)	93.66 (85.33–99)	0.054 ^‡^
Pulse pressure	40 (36–52)	41 (35–50)	0.801 ^‡^
Heart rate	79.52 ± 11.28	80.84 ± 11.24	0.527 ^†^
CIMT	0.06 (0.05–0.07)	0.06 (0.05–0.06)	0.442 ^‡^
Augmentation pressure	6 (4–9)	8.5 (5–11)	**0.045 ^‡^**
Augmentation index	20.32 ± 10.71	25.98 ± 10.21	**0.005 ^†^**
Pulse wave velocity	7.25 (5.9–8.2)	6.3 (5.9–6.9)	**0.012 ^‡^**

Descriptive statistics are presented using mean ± standard deviation for normally distributed continuous variables, median (25th percentile–75th percentile) for non-normally distributed continuous variables and frequency (percentage) for categorical variables. ^†^ Student’s *t* test, ^‡^ Mann–Whitney U test, ^§^ Chi-square test, ^#^ Fisher’s exact test. Statistically significant *p* values are shown in bold. Abbreviations: ALP: alkaline phosphatase, ALT: alanine aminotransferase, APRI: aspartate aminotransferase to platelet ratio index, AST: aspartate aminotransferase, BARD score: body mass index, aspartate aminotransferase/alanine aminotransferase ratio, and diabetes score, CIMT: carotid intima-media thickness, CRP: C-reactive protein, FIB-4: fibrosis-4 index, GH: growth hormone, GGT: gamma-glutamyl transferase, HbA1c: hemoglobin A1c, HDL-C: high-density lipoprotein cholesterol, HOMA-IR: homeostatic model assessment for insulin resistance, IGF-1: insulin-like growth factor-1, LDL-C: low-density lipoprotein cholesterol, MASLD: metabolic dysfunction-associated steatotic liver disease, RDW-CV: red cell distribution width coefficient of variation, USG: ultrasonography, WBC: white blood cell.

**Table 3:** Significant factors independently associated with acromegaly and multivariable logistic regression analysis.

	β Coefficient	Standard Error	*p*	Exp (β)	95% CI for Exp (β)	
Height, cm	0.238	0.112	0.033	1.268	1.019	1.578
Glucose	0.107	0.055	0.049	1.113	1.000	1.240
MASLD fibrosis score	5.722	2.037	0.005	305.607	5.635	16,574.949
Constant	−41.585	20.128	0.039	0.000		

Nagelkerke R^2^ = 0.929. Abbreviations: CI: confidence interval, MASLD: metabolic dysfunction-associated steatotic liver disease.

**Table 4:** Summary of demographics and laboratory measurements with regard to disease activity.

	Disease Activity		
	Active (n = 24)	Biochemical Remission (n = 26)	*p*
Age	46.5 (37–58.5)	50.5 (43–55)	0.984 ^‡^
Sex			
Female	18 (75.00%)	15 (57.69%)	0.321 ^§^
Male	6 (25.00%)	11 (42.31%)	
Height, cm	163 (158–169)	168.5 (162–174)	0.093 ^‡^
Weight, kg	79.5 (72–89)	90.5 (79–100)	0.066 ^‡^
Body mass index, kg/m^2^	29.52 (27.02–33.61)	31.36 (27.75–35.06)	0.404 ^‡^
Obesity	11 (45.83%)	17 (65.38%)	0.269 ^§^
Extreme obesity	2 (8.33%)	1 (3.85%)	0.602 ^#^
Waist circumference, cm	88.25 ± 11.09	94.12 ± 12.55	0.087 ^†^
Hip circumference, cm	102 (96–106.5)	105 (101–110)	0.224 ^‡^
Waist to hip ratio	0.87 ± 0.06	0.89 ± 0.06	0.159 ^†^
Normal	11 (45.83%)	10 (38.46%)	0.810 ^§^
High	13 (54.17%)	16 (61.54%)	
Smoking	4 (16.67%)	2 (7.69%)	0.409 ^#^
Hypertension	8 (33.33%)	9 (34.62%)	1.000 ^§^
Diabetes mellitus	8 (33.33%)	6 (23.08%)	0.623 ^§^
Cardiovascular disease	12 (50.00%)	12 (46.15%)	1.000 ^§^
Type of adenoma			
Macroadenoma	20 (83.33%)	17 (65.38%)	0.261 ^§^
Microadenoma	4 (16.67%)	9 (34.62%)	
Duration of disease, years	8 (5–13.5)	13.5 (4–15)	0.185 ^‡^
IGF-1	292.2 (193.6–556.0)	152.25 (106.7–238.4)	**<0.001 ^‡^**
GH	2.71 (1.88–4.46)	0.36 (0.13–0.91)	**<0.001 ^‡^**
IGF-1, peak	774.03 ± 325.64	680.13 ± 384.89	0.358 ^†^
GH, peak	8.59 (3.70–19.41)	5.11 (1.77–11.24)	0.193 ^‡^
Glucose	103.05 (92.55–123.2)	96.3 (86.1–104)	0.145 ^‡^
Insulin	8.86 (5.76–13.58)	9.67 (6.70–13.10)	0.573 ^‡^
HOMA-IR	2.26 (1.58–3.46)	2.26 (1.55–3.52)	0.884 ^‡^
HbA1c	6.0 (5.6–6.3)	5.75 (5.4–6.2)	0.228 ^‡^
CRP	0.97 (0.40–3.87)	2.10 (1.58–3.44)	0.064 ^‡^
Total cholesterol	203.21 ± 29.13	201.84 ± 40.15	0.892 ^†^
HDL-C	48.26 ± 12.90	47.83 ± 13.62	0.908 ^†^
LDL-C	118.64 ± 30.15	112.87 ± 37.32	0.552 ^†^
Triglyceride	154.05 (121.8–208.75)	206.4 (140.4–270)	0.095 ^‡^
AST	25 (20–27.75)	26.5 (22–31.9)	0.142 ^‡^
ALT	14.5 (12.55–17)	19 (15.6–25)	**0.015 ^‡^**
GGT	14 (11–26)	17 (11–21)	0.877 ^‡^
ALP	75 (60–89)	76 (65–95)	0.466 ^‡^
Albumin	4.04 ± 0.52	4.16 ± 0.34	0.316 ^†^
WBC (×10^3^)	7.15 ± 2.15	6.34 ± 1.41	0.121 ^†^
Neutrophil (×10^3^)	3.68 (3.27–4.83)	3.51 (2.90–4.29)	0.225 ^‡^
Lymphocyte (×10^3^)	2.22 ± 0.55	2.08 ± 0.52	0.367 ^†^
Monocyte (×10^3^)	0.50 (0.39–0.60)	0.48 (0.39–0.50)	0.320 ^‡^
RDW-CV	13.5 (13.0–14.35)	13.5 (12.8–14.3)	0.634 ^‡^
Hemoglobin	12.87 ± 1.43	13.36 ± 1.02	0.170 ^†^
Platelet (×10^3^)	205.58 ± 42.07	199.62 ± 42.14	0.619 ^†^
APRI	0.30 (0.21–0.35)	0.34 (0.27–0.38)	0.085 ^‡^
FIB-4	1.35 (1.15–1.65)	1.49 (1.16–1.93)	0.478 ^‡^
MASLD fibrosis score	0.05 ± 1.33	0.07 ± 1.01	0.942 ^†^
BARD score	3.5 (3–4)	3 (3–4)	0.871 ^‡^
Liver USG			
Normal	5 (29.41%)	1 (7.69%)	0.273 ^¶^
Grade 1	10 (58.82%)	8 (61.54%)	
Grade 2	2 (11.76%)	4 (30.77%)	
Systolic blood pressure	123 (110.5–138.5)	122.5 (115–134)	0.938 ^‡^
Diastolic blood pressure	84.67 ± 13.13	84.46 ± 14.38	0.958 ^†^
Mean arterial pressure	96.16 (89.33–106.50)	94.00 (88.33–108.33)	0.734 ^‡^
Pulse pressure	41.5 (32.5–51.5)	39 (38–43)	1.000 ^‡^
Heart rate	81.00 ± 11.76	75.50 ± 9.31	0.072 ^†^
CIMT	0.06 (0.05–0.07)	0.06 (0.05–0.07)	0.518 ^‡^
Augmentation pressure	6 (4–7)	8 (5–11)	0.151 ^‡^
Augmentation index	19.04 ± 10.42	20.64 ± 10.51	0.595 ^†^
Pulse wave velocity	7.15 (5.6–8.55)	7.3 (6.1–8.1)	0.969 ^‡^

Descriptive statistics are presented using mean ± standard deviation for normally distributed continuous variables, median (25th percentile–75th percentile) for non-normally distributed continuous variables and frequency (percentage) for categorical variables. ^†^ Student’s *t* test, ^‡^ Mann–Whitney U test, ^§^ Chi-square test, ^#^ Fisher’s exact test, ^¶^ Fisher–Freeman–Halton test. Statistically significant *p* values are shown in bold. Abbreviations: ALP: alkaline phosphatase, ALT: alanine aminotransferase, APRI: aspartate aminotransferase to platelet ratio index, AST: aspartate aminotransferase, BARD score: body mass index, aspartate aminotransferase/alanine aminotransferase ratio, and diabetes score, CIMT: carotid intima-media thickness, CRP: C-reactive protein, FIB-4: fibrosis-4 index, GH: growth hormone, GGT: gamma-glutamyl transferase, HbA1c: hemoglobin A1c, HDL-C: high-density lipoprotein cholesterol, HOMA-IR: homeostatic model assessment for insulin resistance, IGF-1: insulin-like growth factor-1, LDL-C: low-density lipoprotein cholesterol, MASLD: metabolic dysfunction-associated steatotic liver disease, RDW-CV: red cell distribution width-coefficient of variation, USG: ultrasonography, WBC: white blood cell.

**Table 5:** Correlation analyses between IGF-1, GH, and MASLD markers in patients with acromegaly.

		IGF-1	GH	IGF-1, Peak	GH, Peak
APRI	r	0.132	0.079	0.066	0.090
	*p*	0.322	0.554	0.623	0.501
FIB-4	r	0.042	0.140	−0.070	−0.104
	*p*	0.754	0.294	0.599	0.435
MASLD fibrosis score	r	−0.001	0.020	0.027	−0.048
	*p*	0.993	0.882	0.840	0.721
BARD score	r	−0.081	−0.162	0.078	−0.112
	*p*	0.547	0.225	0.563	0.403
Liver USG	r	−0.100	−0.096	0.154	−0.015
	*p*	0.556	0.571	0.363	0.928

Abbreviations: APRI: aspartate aminotransferase to platelet ratio index, FIB-4: fibrosis-4 index, GH: growth hormone, IGF-1: insulin-like growth factor-1, MASLD: metabolic dysfunction-associated steatotic liver disease, BARD score: body mass index, aspartate aminotransferase/alanine aminotransferase ratio, and diabetes score, USG: ultrasonography, r: correlation coefficient.

## Data Availability

The datasets generated and/or analyzed during the current study are not publicly available but are available from the corresponding author on reasonable request.
